# Binder-Free Electrodes and Their Application for Li-Ion Batteries

**DOI:** 10.1186/s11671-020-03325-w

**Published:** 2020-05-18

**Authors:** Yuqiong Kang, Changjian Deng, Yuqing Chen, Xinyi Liu, Zheng Liang, Tao Li, Quan Hu, Yun Zhao

**Affiliations:** 1 Shenzhen Key Laboratory on Power Battery Safety Research and Shenzhen Geim Graphene Center, Tsinghua Shenzhen International Graduate School, Shenzhen, 518055 China; 2 Hoffmann Institute of Advanced Materials, Shenzhen Polytechnic, Shenzhen, 518055 China; 3 Department of Chemistry and Biochemistry, Northern Illinois University, DeKalb, IL 60115 USA; 4 Department of Materials Science and Engineering, Stanford University, Stanford, CA 94305 USA; 5 Changsha Nanoapparatus Co., Ltd, Changsha, 410017 China

**Keywords:** Lithium ion batteries, Binder-free electrode, Fabrication method, Flexible

## Abstract

Lithium-ion batteries (LIB) as energy supply and storage systems have been widely used in electronics, electric vehicles, and utility grids. However, there is an increasing demand to enhance the energy density of LIB. Therefore, the development of new electrode materials with high energy density becomes significant. Although many novel materials have been discovered, issues remain as (1) the weak interaction and interface problem between the binder and the active material (metal oxide, Si, Li, S, etc.), (2) large volume change, (3) low ion/electron conductivity, and (4) self-aggregation of active materials during charge and discharge processes. Currently, the binder-free electrode serves as a promising candidate to address the issues above. Firstly, the interface problem of the binder and active materials can be solved by fixing the active material directly to the conductive substrate. Secondly, the large volume expansion of active materials can be accommodated by the porosity of the binder-free electrode. Thirdly, the ion and electron conductivity can be enhanced by the close contact between the conductive substrate and the active material. Therefore, the binder-free electrode generally exhibits excellent electrochemical performances. The traditional manufacture process contains electrochemically inactive binders and conductive materials, which reduces the specific capacity and energy density of the active materials. When the binder and the conductive material are eliminated, the energy density of the battery can be largely improved. This review presents the preparation, application, and outlook of binder-free electrodes. First, different conductive substrates are introduced, which serve as carriers for the active materials. It is followed by the binder-free electrode fabrication method from the perspectives of chemistry, physics, and electricity. Subsequently, the application of the binder-free electrode in the field of the flexible battery is presented. Finally, the outlook in terms of these processing methods and the applications are provided.

## Introduction

**Fig. 1:**
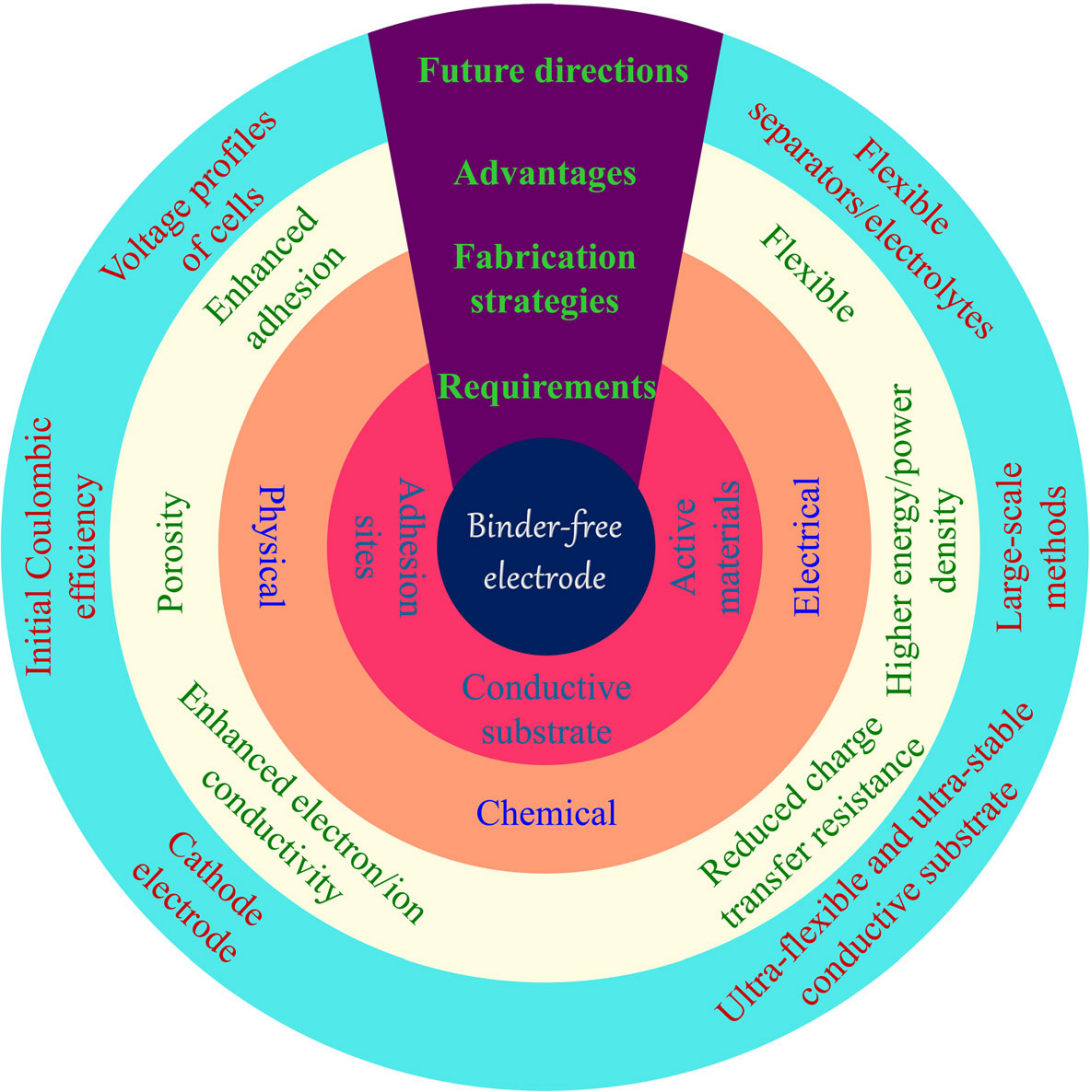
The requirements, fabrication methods, advantages, and future development for binder-free electrode

The energy crisis and environmental issues have driven the development of renewable energy and new environmentally friendly energy storage systems. Because of the intermittent problem of renewable energy sources such as wind energy, water energy, and solar energy, batteries are considered to be important energy storage systems [1–3]. There is an increasing demand for reliable and efficient energy storage devices. Lithium-ion batteries (LIBs) have attracted much attention due to their high energy and power density, high cell voltage, wide operating temperature range, and long cycle life [4]. Nowadays, the traditional process of the battery preparation uses a polyvinylidene fluoride (PVDF) as a binder to fix the conductive agent and the active materials on the current collector by a coating method [5, 6]. With the demand for the LIBs with higher capacity and smaller size, both the development of active materials with high specific capacity and the reduction of inactive materials in the cell are important. The methods to reduce the inactive materials are as following. Firstly, the traditional binder can be replaced by the conductive binder, for example, pyrene-based polymer and polyfluorene-conjugated polymer. These polymers are naturally conductive, and their side-chain or backbone is modified to increase the adhesion [7–10]. The conductive binder serves as conductive agent. Therefore, the use of inactive carbon in the cell can be reduced. However, the weak interfacial interaction between these binders (both PVDF and new developed binders) and active materials (metallic oxide, Si, Sn, Li, S, etc.) results in the particles self-aggregate or/and isolation from current collector. Therefore, these new materials with high capacity show reduced battery performance [11–15]. Secondly, advanced conductive substrates, for example, carbon cloth, graphene, and Ni foam, are investigated, where active materials can be anchored on the special adhesion sites of substrates. The adhesions between active materials and substrates are achieved by strong chemical and/or physical bonding, which significantly improves the integrity of electrodes. Moreover, this process potentially removes both binder and conductive carbon additives. Therefore, the energy density can be largely improved [16, 17] (Fig. 1).

Great deal of research has demonstrated the numerous advantages of binder-free electrodes [18–21]. By immobilizing the active materials onto the corresponding electron-conductive substrate, the interfacial problem of binder and active materials can be resolved due to the absence of organic binder covering on the active materials surface [22, 23]. Active materials firmly adhere on the conductive substrate, which highly improves the electron conductivity. The properties of supporting materials, for example, porous structures, facilitate electrolyte penetration, and ion diffusion [24]. Besides, the large surface area has the benefit for the full usage of active materials and the transportation of Li-ions. Moreover, the active material is generally uniformly anchored on the conductive substrate, which can effectively prevent the agglomeration of the nanoparticles and reduce the volume expansion during the repeated cycling process. The binder-free electrodes generally show high Li^+^ and electron conductivity, decent electrolyte wettability and large volume expansion space, and strong bonding strength. Therefore, the binder-free electrodes exhibit better capacity, cycling, and rate performances than the PVDF/active materials/carbon black system. Specifically, the cycle life of the novel nanomaterials has been increased from dozens of cycles to hundreds of cycles, with a high current density of ~ 10 A g^−1^.

The conductive substrate as a carrier for the active material is the basis of the binder-free electrode. The conductive matrix needs to have suitable sites for growing active materials, and its mechanical properties play a decisive role in its application. For applications of the electrodes in wearable and flexible electronic devices, conductive substrates need to be able to be bent or even folded multiple times. This is difficult to achieve in conventional electrodes fabricated by the slurry process. The main reason is that the active material is separated from the current collector during the bending process, resulting in the deactivation of the battery. Growing the active materials directly on a flexible network provides a strong interaction and leads to robust electrodes maintaining the high energy density. These flexible substrates mainly include metal foam, carbon cloth, and free-standing films of carbon materials [25].

This review aims to provide an overview of preparation, application, and outlook of binder-free electrodes for LIBs. Our goal is to highlight the recent development and improvement of binder-free electrodes [26]. The doctor blade casting and infiltration methods, which are undoubtedly important to the field of LIBs, will not be included. First, we introduce the different conductive substrates, which serve mainly as carriers for the active materials. We follow with a presentation on the binder-free electrode fabrication method from the perspectives of chemistry, physics, and electricity. Subsequently, the application of the binder-free electrode in the field of the flexible battery is presented. Finally, the key issues concerning these preparation methods and their applications are prospected.

## Conductive Substrate

The conductive substrate is the current collector with good electronic conductivity. Therefore, the material is generally composed of metal or carbon material. Due to manufacturing limitations, metal current collectors are typically fabricated into films, meshes [27], and foams [28]. Metal products are generally rigid and are not easily recovered after deformation; therefore, they are only suitable for high energy density batteries of the same configuration as slurry-based batteries. Copper and aluminum are used as a negative and positive current collector, respectively, due to the different oxidation resistance [29]. Metal foam has the advantages of light weight, large area, three-dimensional structure, and the like, which is often used for binder-free electrodes [30].

Carbon materials originate from a range of sources and are very flexible in their preparation [31]. These materials can be derived from a wide variety of biological materials in nature, as well as chemically prepared carbon nanotubes, graphene, and porous carbon structures from organic materials [17, 32]. Compared with metal, some kinds of carbon materials are lighter in weight and have great flexibility (flexible, foldable, etc.). Carbon cloth is increasingly applied in energy storage due to its excellent electrical conductivity and flexibility [33].

## Chemical Methods

### Thermal Treatment

**Fig. 2:**
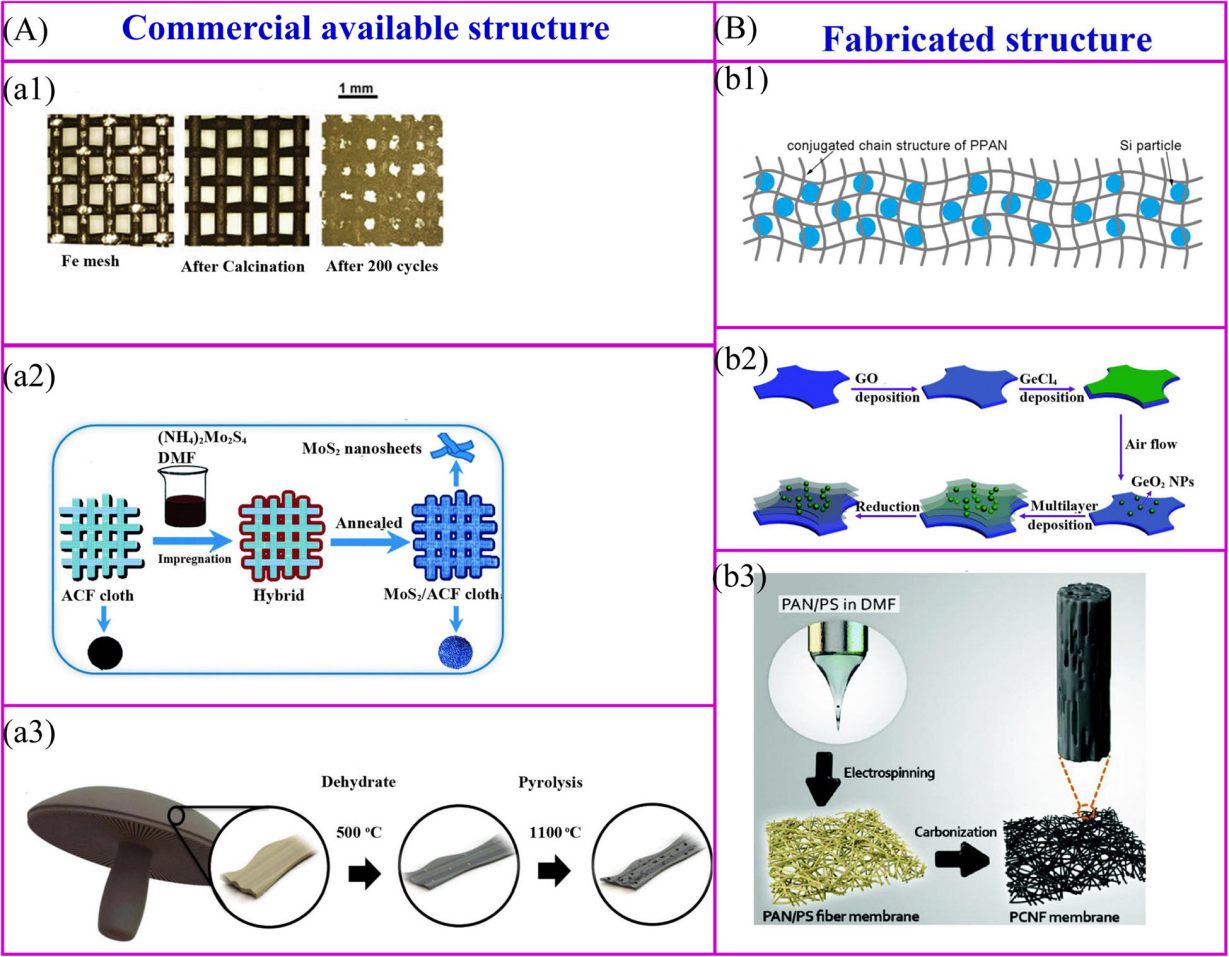
Thermal treatment for the commercially available structure (**a**) and fabricated structure (**b**). **a1** Metallic oxide nanoparticles can be obtained on the surface of an metal structure via a simple thermal oxidation progress [34]. **a2** The active materials can be synthesized on the surface of conductive structure by thermal treatment [35]. **a3** Biomass can be carbonized to achieve the carbon architecture [32]. **b1** The mixture of polymer and active materials can be carbonized to achieve the binder-free electrode [36]. **b2** Hierarchical structure can be obtained by multiple processes [37]. **b3** Binder-free electrode can be obtained by thermal treatment the electrospinning membrane [38]

Thermal treatment is one of the common methods of preparing a binder-free electrode. This method is to change the physical and chemical properties of the material by means of heating and cooling process. After heat treatment, the inorganic salt is converted to the corresponding metal oxide, and the polymer will dehydrate to form a carbon conductive structure (Fig. 2). For the preparation of binder-free electrodes, thermal treatment is generally used to immobilize the active material or to construct a self-supporting backbone.

The commercially available structure is utilized as supporting skeleton to immobilize active materials. These materials consist of metal meshes, carbon fibers, commercial sponges, and biological derivatives and commercial sponge [39], etc. (Fig. 2a). Metallic oxide nanoparticles can be synthesized on the surface of metal current collectors via simple thermal oxidation progress [34] (Fig. 2a1). Without any further treatment, these current collectors can be used directly as supporting materials for binder-free LIBs. Iron mesh-supported Fe_2_O_3_ shows a very high discharge capacity of 1050 mAh g^−1^ after 200 cycles. Thermal treatment of the conductive membrane with precursor’s solution of active materials is a widely developed method for the fabrication of binder-free electrodes (Fig. 2a2). A representative example is that the ultrathin MoS_2_ nanosheets coated at the surface of active carbon fiber (ACF) cloth can be fabricated by immersing in the (NH_4_)_2_MoS_4_ solution followed by annealing. The electrochemical performances are demonstrated that the discharge capacity of 971 mAh g^−1^ is achieved at a current density of 100 mA g^−1^ [35]. Thermal treatment of biomass materials is a simple method for preparation of binder-free electrode. Ozkan and coworkers carbonized the portobello mushroom as binder-free LIBs anodes (Fig. 2a3) [32]. At high temperature, the structure of biomass materials can be remain, and the naturally presented heteroatoms and metal ions can dope in the carbon materials, which increases the electrochemical performances such as electron conductivity and capacity.

The polymer is the main material for constructing the self-supporting skeleton of the binder-free electrode, and the skeleton structure is determined by the polymer and its preparation method (Fig. 2b) [40]. Firstly, for the common polymers, pyrolysis of polymer-active materials composite film at 550 °C can prepare binder-free electrode (Fig. 2b1) [36]. The Si/SiO_x_/PAN composite electrode is prepared by this method [41, 42]. After annealing, the polyacrylonitrile (PAN) can be converted to N-doped conductive structure, and the carbon network not only stabilizes the SEI and accommodate the volume changes but also provide good flexibility and mechanical strength for the electrode. Similarly, Si/rGO electrode can be obtained by casting Si, reduced graphene oxide (rGO) and polyvinylpyrrolidone (PVP) suspension onto nickel foam followed by an annealing process [43]. Secondly, the layer-by-layer (LBL) process is an attractive way to make complex structures and nanomaterials. An electrode with multiple layers can be fabricated by immersing the Ti foil in poly (diallyldimethylammonium chloride) (PDDA) solution, graphene oxide (GO) suspension, PDDA solution, and aqueous H_3_PMo_12_O_40_ at certain cycles, then followed by thermal treatment at 500 °C [44]. Such LBL method can be applied to prepare a binder-free electrode on a large scale. This kind of method is suitable for making mesoporous anatase TiO_2_/nickel foam [45], MoS_2_ nanosheet/ACF, and multilayer GeO_2_/rGO (Fig. 2b2) [37, 46]. Last, binder-free electrodes can be fabricated by encapsulation of active materials into polymers and then fabrication to novel nanostructure (Fig. 2b3). Flexible hierarchical nanofibers mats can be synthesized by electrospinning and subsequent thermal treatment.

There are many merits for commercially available and fabricated structure. The active material is coated on the surface of the commercially available structure while the fabricated structure acts as a container to encapsulate the active material. In contrast to the encapsulation of active materials, the surface coating makes the more contact of active materials and electrolyte. Therefore, it results in the better rate performance but lower initial Coulombic efficiency and poor cycling performance.

### Hydrothermal Treatment

The hydrothermal method is widely used in different disciplines in past several decades. Currently, this technique has made great effort in terms of mechanisms interpretation and material fabrication. For the hydrothermal process, metal ions are dissolved in the solution which afterwards forms a supersaturated solution at high temperature and pressure. During this process, crystal growth occurs at the nucleation point of the substrate. Comparing to the aggregated particles prepared by thermal treatment, the hydrothermal method can produce high-pure, uniform, monodispersed, and controllable nanoscale materials under mild conditions. The hydrothermal process of fine nanostructure has attracted widespread attention in energy storage materials.

**Fig. 3:**
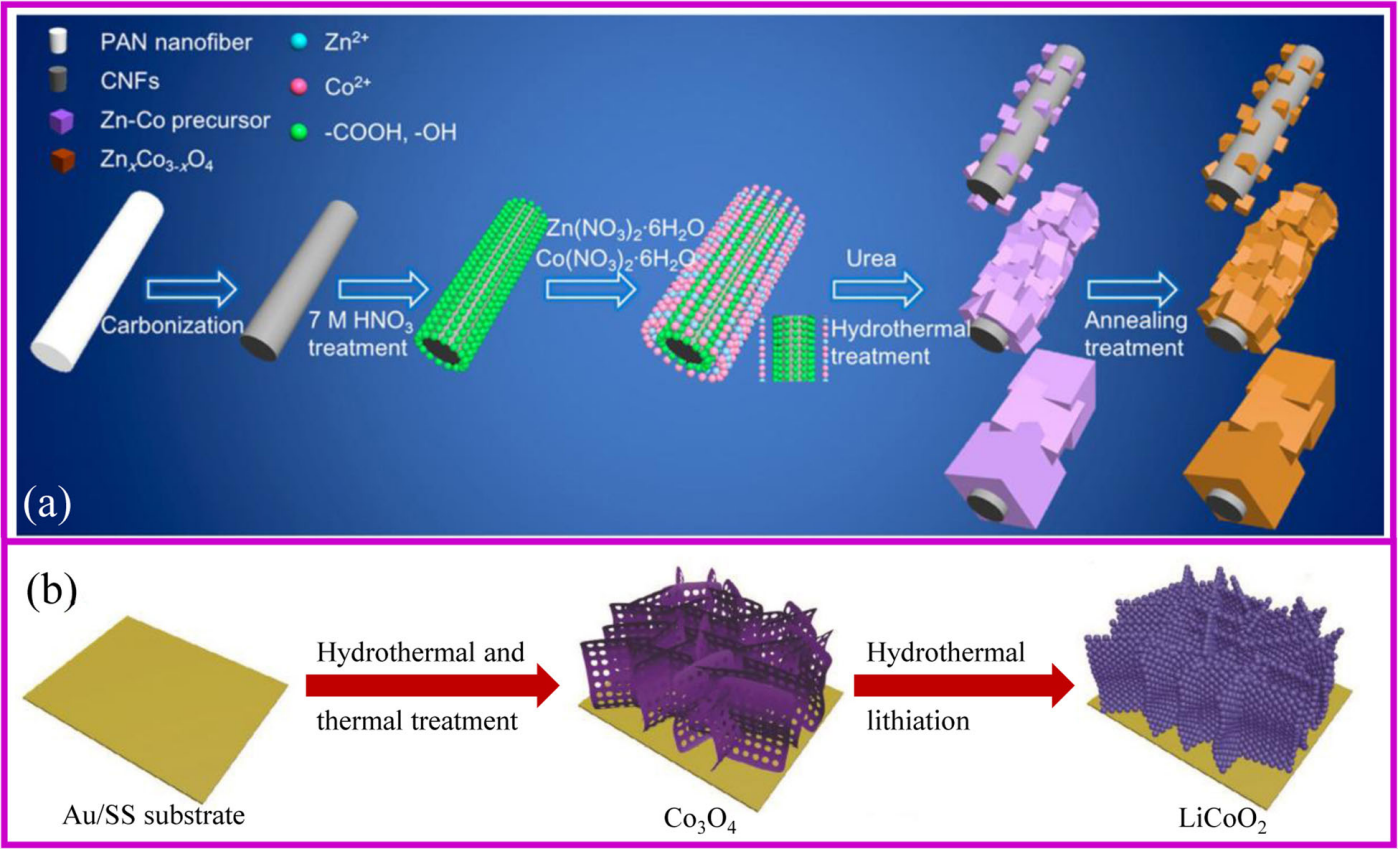
**a** The scheme of ZnCoO_x_/CNF composite fabrication [47]. **b** The fabrication of cathode electrode using hydrothermal method [48]

An overall synthetic process for the preparation binder-free electrode using hydrothermal method is similar to the procedure described in Fig. 3a. Supporting materials are first obtained. If the supporting materials are smooth with limited nucleating points, the deposition of active materials on their surface would be prohibited. Generally, the carbon cloth needs acidic or thermal treatment to become more hydrophilic. Besides, the pH of the solution should be adjusted by adding a suitable precipitant to promote the growth of the precursor on the surface of the substrate. The obtained materials are thermally treated to obtain the desired composite while maintaining the nanostructure. Hu, Zhang, and coworkers reported a scalable method for the preparation of Zn_x_Co_3-x_O_4_ nanocubes/CNFs (carbon nano-fibers, CNFs). The cube’s size can be adjusted by the pH applied in the hydrothermal process [47].

The hydrothermal method can fabricate single and multiple component [49]. Many morphologies of binder-free electrodes have been developed, such as TiO_2_ nanorods on carbon nanotube (CNT) scaffold [50], Fe_3_O_4_ nanoparticles, NiO nanocones, Ni(OH)_2_ nanosheets and Fe_3_O_4_/Ni/C nanoplates grown on Ni foam [51–54], MnO_2_ nanoflakes on graphene foam [55], and FeF_3_·0.33H_2_O flower-like arrays on carbon fiber [56]. Li and coworkers grew NiCo_2_S_4_ nanotube arrays showing unique 3D structure, in which NiCo_2_S_4_ nanotubes show 5 nm in length and 100 nm in width [57]. Porous NiCo_2_O_4_ nanoneedles grown on 3D graphene network can be obtained by using NiCl_2_·6H_2_O and CoCl_2_·6H_2_O as the precursors [58]. These nanostructures homogeneously distribute on the conductive substrate. Therefore, these composites not only facilitate the electron transfer and accommodate the volume changes of the active materials during discharge/charge process, but also improve the electrochemical properties with high capacity, high rate capability, and cycling stability for LIBs. Specifically, Fe_3_O_4_ nanoparticle@Ni foam showed a reversible capacity of 543 mA h g^−1^ at the current density of 10 C after more than 2000 cycles [51]. NiO arrays@Ni foam can deliver a capacity of 969 mAh g^−1^ at the current density of 0.5 C and still remain about 605.9 mAh g^−1^ at 10 C [52].

It is worth noting that hydrothermal method is a good strategy to achieve the lithiation of metal oxides for cathode materials. Conventional lithiation requires uniform mixing of the precursor with Li salt, which is every difficult to obtain the desired binder-free electrodes. Hydrothermal lithiation is a solution method that does not require the treatment of the precursor, so it is one of the attractive methods for fabricating the binder-free cathode electrode. In 2018, Xia et al. prepared the porous LiCoO_2_ binder-free cathode with Au-coated stainless steel as substrates by the hydrothermal lithiation of Co_3_O_4_ precursor (Fig. 3b) [48]. This electrode shows excellent rate and cycling performances with a capacity of 104.6 mA h g^−1^ at 10 C rate and the capacity retention of 81.8% after 1000 cycles.

### Chemical Bath Deposition

**Fig. 4:**
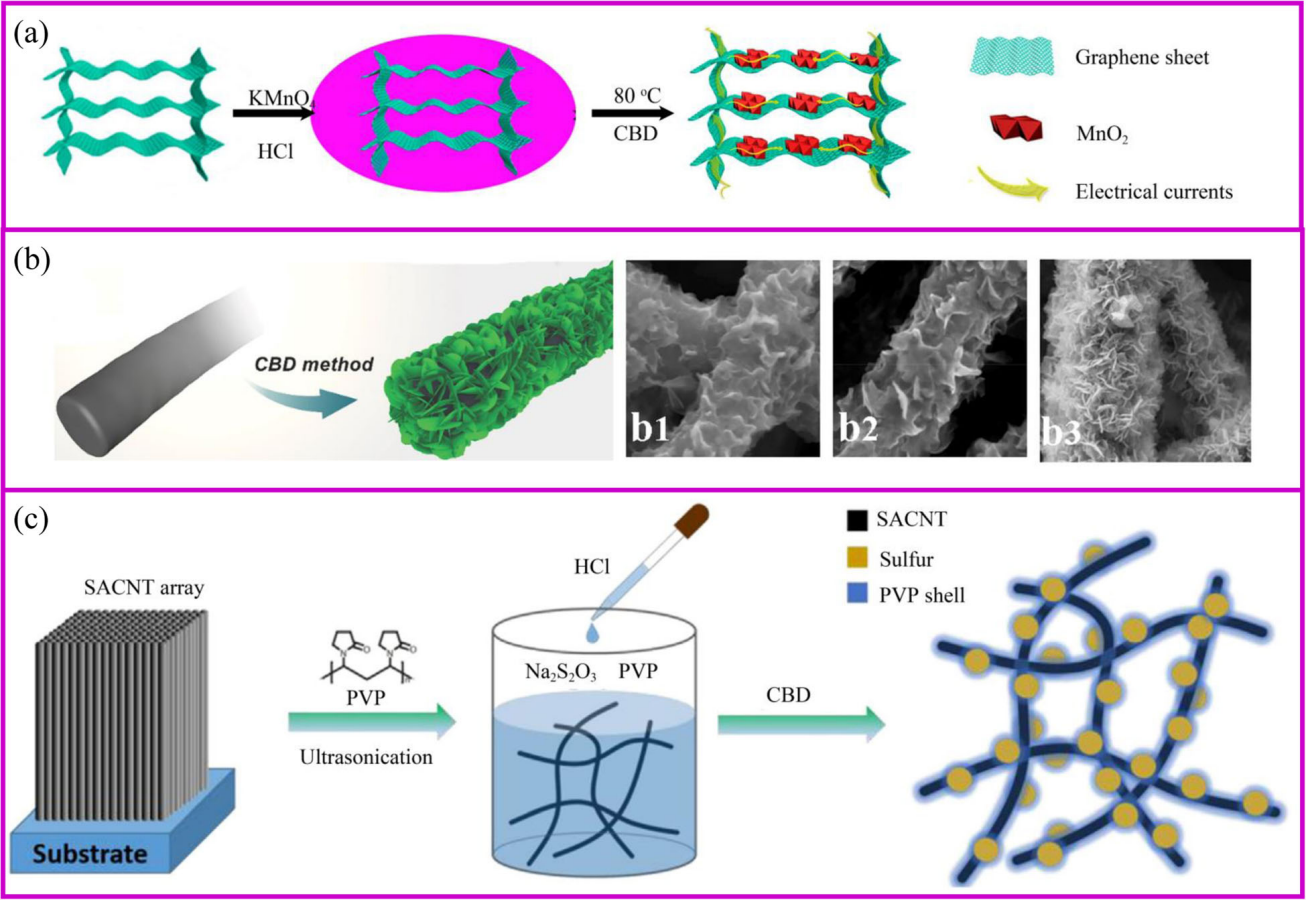
**a** Schematic illustration of the preparation of 3D graphene/MnO_2_ hybrid, and illustrations of electrons transfer on 3D graphene/MnO_2_ hybrid [59]. **b** CBD method for the fabrication of CNF@Ni(OH)_2_ [60]. **b1–3** Different hybrid membranes with increase of concentrations of Ni(NO_3_)_2_ solution. **c** Schematic of the synthesis procedure for the PVP@S-SACNT composite [61]

Chemical bath deposition (CBD) is a process of in situ growth of active materials on the substrate through a chemical reaction. Comparing with the hydrothermal method, this synthesis method is easy to scale up and allows nanomaterials to grow at low temperature and ambient pressure without using special equipment. Besides, the CBD and hydrothermal method grow materials on the surface of substrates via a similar mechanism, so the requirements for the substrates are very similar. As the same to the procedure as shown in Fig. 4a, the precursor of active materials would nucleate and grow by adjusting the pH and temperature of reactions. For example, 3D graphene/MnO_2_ hybrids are prepared by the presence of 3D graphene aerogel in the acidic KMnO_4_ solution [59].

The morphology of active materials is influenced by supporting materials, reacting time, and precursor concentration (Fig. 4b). The substrate determines the initial nucleation sites. For example, the morphology of MnO_2_ is nanosheet and nanoparticle on the substrate of graphene and CNTs, respectively [62, 63]. In addition, the morphology of active materials on the supporting material is influenced by precursor concentration. For example, thin Ni(OH)_2_ nanosheets begin to form and grow perpendicularly on the surface of nanofibers at low Ni(NO_3_)_2_ concentration (Fig. 4b) [60]. However, with the increase of Ni salt concentrations, a thick layer of Ni(OH)_2_ nanosheets is gradually formed, which may be attributed to the rapid and homogeneous nucleation of Ni(OH)_2_. Therefore, the morphology of active materials on the supporting material can be various, such as particles [64], sheath, nanosheet [65], and nanowires [66, 67]. Similar to the electrode prepared by hydrothermal method, the porous and conductive architecture with nanoscale materials can provide continuous channels for rapid diffusion of lithium ions and efficient transport of electrons for fast lithiation/delithiation.

Sulfur, a very promising cathode material, can be synthesized by CBD under mild conditions. The sulfur material is based on a simple reaction between Na_2_S_2_O_3_ and acids in an aqueous solution at room temperature. The process is simple and environmentally benign. When a suitable template or surfactant is applied, the special nano-sulfur structure can be obtained [68]. When the conductive materials can absorb S_2_O_3_^2-^, a great quantity of sulfur is generated at the interphases. Graphene modified by phenyl sulfonated functional groups allows the uniform deposition of sulfur via an in situ redox reaction [69]. The binder-free PVP-encapsulated sulfur electrode is prepared by the in situ immobilize the sulfur nanoparticles onto the conductive network (Fig. 4c). PVP is an amphiphilic polymer with a hydrophobic alkyl chain and hydrophilic amide groups that can be used as a dispersing agent. When sulfur starts to form after adding acid into the solution, the hydrophobic nature of PVP makes it preferentially coat onto the S surface forming a dense layer to protect polysulfides dissolution [61].

### Chemical Vapor Deposition

Chemical vapor deposition (CVD) is a chemical reaction in which a gaseous substance deposits on the surface of a hot substrate. This method can produce the uniform film on the three-dimensional structure and nanowires with the assistance of catalysts. The CVD process consists of three steps: (1) diffusion and absorption of the reaction gases on the surface of the hot substrate, (2) reactions of the gases at the active site to form a coating material, and (3) exhaust of the generated gas. By controlling the temperature, pressure, gases ratio and type, the desired coating material can be obtained.

CVD method can directly grow active materials. An impressive example corresponding to the CVD process was reported by Tay and coworkers [70]. 3D nickel foam/CNTs composite is synthesized with nickel foam as the substrate and ethanol as both precursor and carbon source. The obtained CNTs serve as substrates for the deposition of NiO nanosheet growth. Amorphous FeVO_4_ nanosheet arrays can directly grow on a flexible stainless steel substrate with VCl_3_ as the precursor. It can deliver reversible capacities of 601 mAh g^−1^ and 453 mAh g^−1^ at the high current density of 8 C and 15 C, respectively [71].

Surface layers prepared by CVD also serve as protective interfaces between the electrode and the electrolyte. Yang and coworkers used ethylene as the carbon precursor to coat active materials through a CVD process, which not only improves the stability of the structure but also forms an excellent electronic conductive network. The Si nanowires with a carbon coating layer show a good rate performance [72, 73]. In 2016, Cui et al. showed the porous materials with a thin layer of lithiophilic materials prepared by CVD method can serve as the scaffold promoting the uniform deposition of Li-ion [74]. This material shows a stable cycling performance with a small overpotential even at a high current density of 3 mA/cm^2^ during charge and discharge processes.

CVD method is one of the main strategies for the fabrication of advanced Si materials. Silicon is the most promising anode material for next generation LIBs due to the highest specific capacity of 4200 mAh g^−1^ and low operation voltage [75]. However, silicon suffers from huge volume changes, which leads to continuous formation of solid electrolyte interface (SEI), pulverization, and capacity fading during cycling processes [76]. In general, advanced silicon materials can be prepared by post-treatment of silicon particles or by reduction of silicon dioxide. CVD is desirable way to prepare thin film or nanowire silicon by reducing or pyrolyzing high-purity silane or silane substitutes. In 2008, Cui et al. used CVD method to synthesize silicon nanowires (Si NWs) on the stainless steel with Au nanoparticles as catalysts and successfully applied it as anode for LIBs [77]. Silicon nanowires with a diameter of about 89 nm can accommodate 400% volume change without cracking. In addition, the nanowires are directly grown on the current collector, and all nanowires actively contribute to the capacity. Due to the nanostructure, the entire porous electrode has a very large specific surface area and thus has excellent ion conduction. The nanowire silicon material can achieve a theoretical capacity of almost 4200 mAh g^−1^ for the first time at C/20 rate. Although the diameter of nanowires increased from 89 to 141 nm after cycling process, the overall structure remained intact. The growth of Si is controlled by the catalysts. The stainless steel can also act as catalysts for the Si film formation. However, the formation of Si layers on the current collector can cause severe stress between the Si layer and the collector. The growth of Si can be interfered at a certain step by controlling the active seeds. For example, the chemically stable graphene or metal Ge surface with Au or Sn nanoparticles can serve as seeds for Si NWs growth [78, 79].

### Atomic Layer Deposition

**Fig. 5:**
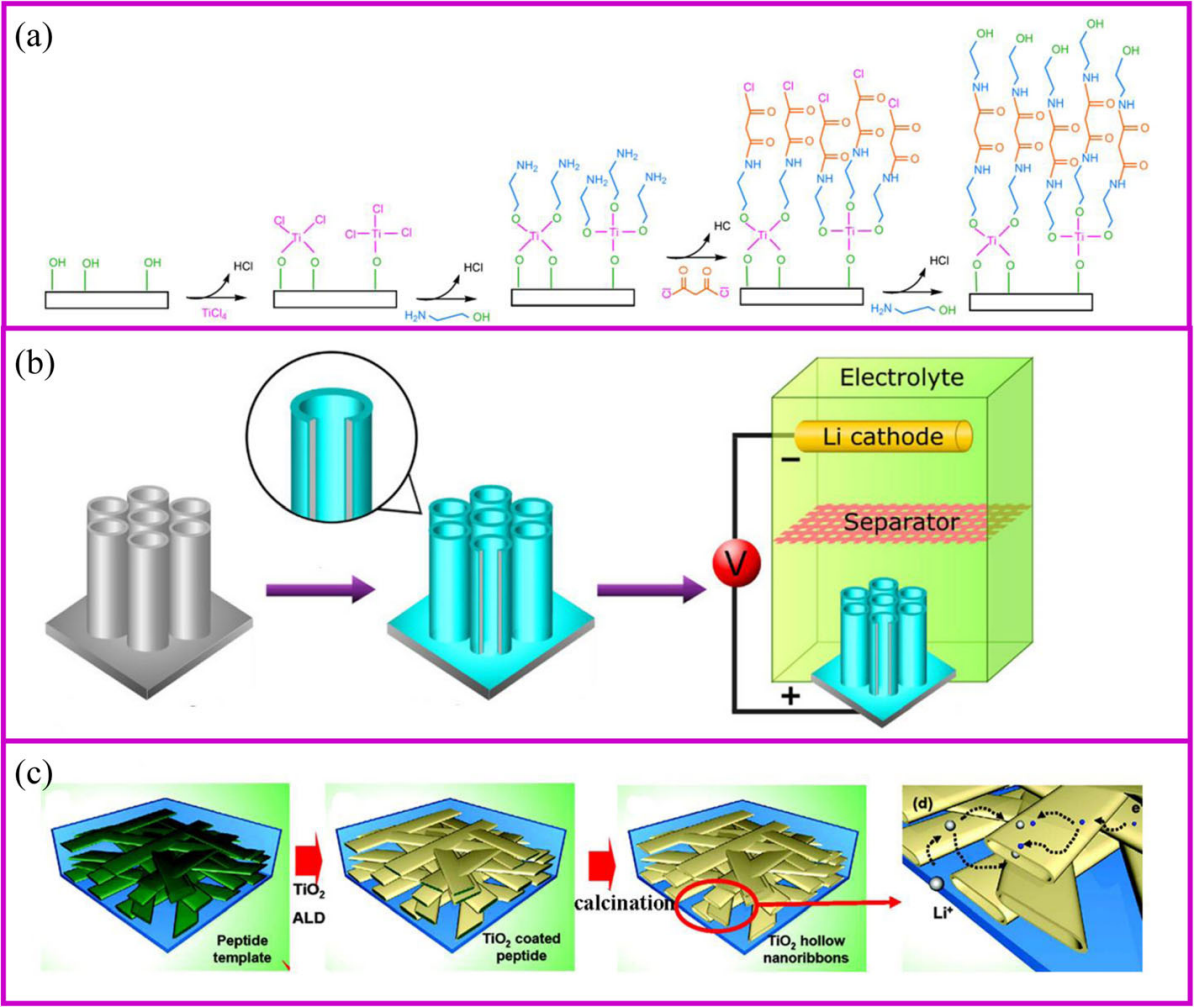
**a** The ALD technique mechanism [81], and two examples for **b** surface coating [82] and **c** active materials fabrication [83]

The atomic layer deposition (ALD) method is a vapor-phase, self-limiting, and layer by layer deposition, which is similar to the CVD. This method can produce nanoscale and controllable thin films in an atomic layer-by-layer deposition. Therefore, the process should consist of at least two different precursor gases, which can react with each other [80]. During the ALD process, the first gas is introduced into the pipe furnace and reacts with the substrate to form a coating layer with active groups. After the first gas is fully emitted, the second gas is introduced to react with the first layer (Fig. 5a) [81]. By repeating this process, different coating layers can be achieved. The coating film by ALD is mainly influenced by the substrate, gases precursors, temperature, etc. Compared with traditional thin-film deposition method, ALD can precisely control the coating thickness across the substrate by chemical reactions, and the coating layers are not only pinhole-free, dense, and uniform but also conformal even when deposited on complex 3D structures. These features of ALD present it as a great choice for nanotechnology and materials.

ALD prepared electrodes generally have good electrochemical properties. TiO_2_ is the most investigated electrode materials (Fig. 5b, c) [84]. Recently, SnO_2_ [85], MoS_2_ [86], etc. are prepared and successfully used as active material for LIBs (Fig. 5c) [87, 88]. Because ALD is a vapor-phase synthesis method, it can coat a uniform layer with controllable thickness on the surface or inside the pores of materials. Kang and coworkers [83] demonstrated that nanoribbons as active materials in the electrodes allows the electrolyte to be immersed inside the material, thereby greatly increasing the diffusion rate of lithium ions. With the assistance of template, the hollow space of the nanoribbons can be synthesized by ALD with the tunnel size of nearly 100–200 nm in width and 20–50 nm in height. It allows the electrolyte to easily wet the hollow space. The rate performance of TiO_2_ nanoscale network has increased at least five times at 5 C compared to that of 100 nm-TiO_2_ nano-powder. Biener et al. coated porous electrode with TiO_2_ layers. It is found that the material with thinner coating layer shows better rate performance. When the TiO_2_ layer thickness increased from 2 to 7 and 20 nm, the capacity decreases from 227 to 214 and 157 mAh g^−1^, respectively [89].

The most general application of ALD in electrochemical storage is to protect the surface stability of electrodes to enhance the electrochemical performance [90]. The uniform Al_2_O_3_ coating on TiO_2_ nanotubes for LIBs is the most representative example of surface protection (Fig. 5a) [82]. The coating thickness of the Al_2_O_3_ layer onto the TiO_2_ nanotube can be controlled by ALD from 0.2, 1 to 10 nm according to the repeated cycles. The 1 nm coating Al_2_O_3_ layer can suppress the SEI formation and undesirable side reactions, which greatly improves the capacity. In addition, Al_2_O_3_ as an artificial layer can participate in the formation of SEI with Li–Al–O groups, which are great ionic conductor. Therefore, the Li-ion conductivity in improved and great rate performance can be achieved. Noked et al. demonstrated the 14 nm Al_2_O_3_ layer can effectively improve the stability of lithium metal interface by avoiding the reactions with electrolyte, cathode shuttles, etc. [91]. Comparing with the bare lithium metal anode, the ALD-protected anode can significantly improve cycling performance.

## Electrical Methods

### Electroplating

Electroplating is a versatile technique that functions to improve the surface properties of materials or to prepare nanoscale structures. The deposition mechanism is that in the case of an applied electric field, the ions move to the positive electrode and are reduced on the surface of the substrate to form a film. The thickness of film is controlled by the current density and time. Through post-treatment, the metal film can be oxidized to the corresponding metal oxide.

Template synthesis is the most popular method for preparing nanostructures of various materials using electroplating in LIBs. Chen, Xia, and coworkers obtained porous CoO semisphere arrays using the polystyrene as the template [92]. Yan, Tong, and coworkers demonstrated that CoO can coat on the surface of ZnO nanorod arrays by electroplating method. The ZnO template can be removed by treating the obtained electrode at KOH solution [93].

Electroplated surface layers also serves as a protective interfaces between the electrode and the electrolyte. Cu/TiO_2_ NT/Ti electrode can be prepared via electroplating Cu on TiO_2_ NT/Ti film. The prepared materials display a much higher discharge capacity, cycle stability, and Li^+^ diffusion coefficient than bare TiO_2_NT/Ti electrode [94]. Mulder et al. designed a 3D Ni honeycomb current collector for stable Li metal anode [95]. By controlling the porosity of Ni material with polyethylene glycol as an additive, the Li plating/stripping performance can prolong to 300 and 200 cycles at 0.5 mAh cm^−2^ and 1.0 mAh cm^−2^, respectively, at 1.0 mA cm^−2^.

### Anodization

Anodization is a well-established technique for modifying a layer on the metal surface. Generally, the metal surface can be thermal treated to form the corresponding oxide protective layer. However, this heating process often carries out at a high temperature, which changes the material structure and properties. Therefore, it is necessary to develop a low temperature method. Anodization refers to a technique in which a metal material is oxidized and precipitated in the electrolyte solution by applying an anode current at room temperature. Anodization is popular because of its controllable structure, economical, and large-area preparation.

Li et al. firstly reported the porous Fe_3_O_4_ thin film as anode material cycled about 100 cycles at the 0.1 C [96]. Subsequently, TiO_2_ [97], NiO [98], WO_3_ [99], CuCl nanoparticles [100], etc. were prepared and showed decent cyclic stability, good ion and electron conductivity, and enhanced capacity. The NiO@Ni foam can deliver a reversible capacity up to 705.5 mAh g^−1^ and 548.1 mAh g^−1^ at a current density of 1 A g^−1^ and 2 A g^−1^, respectively.

### Electrophoretic Deposition

**Fig. 6:**
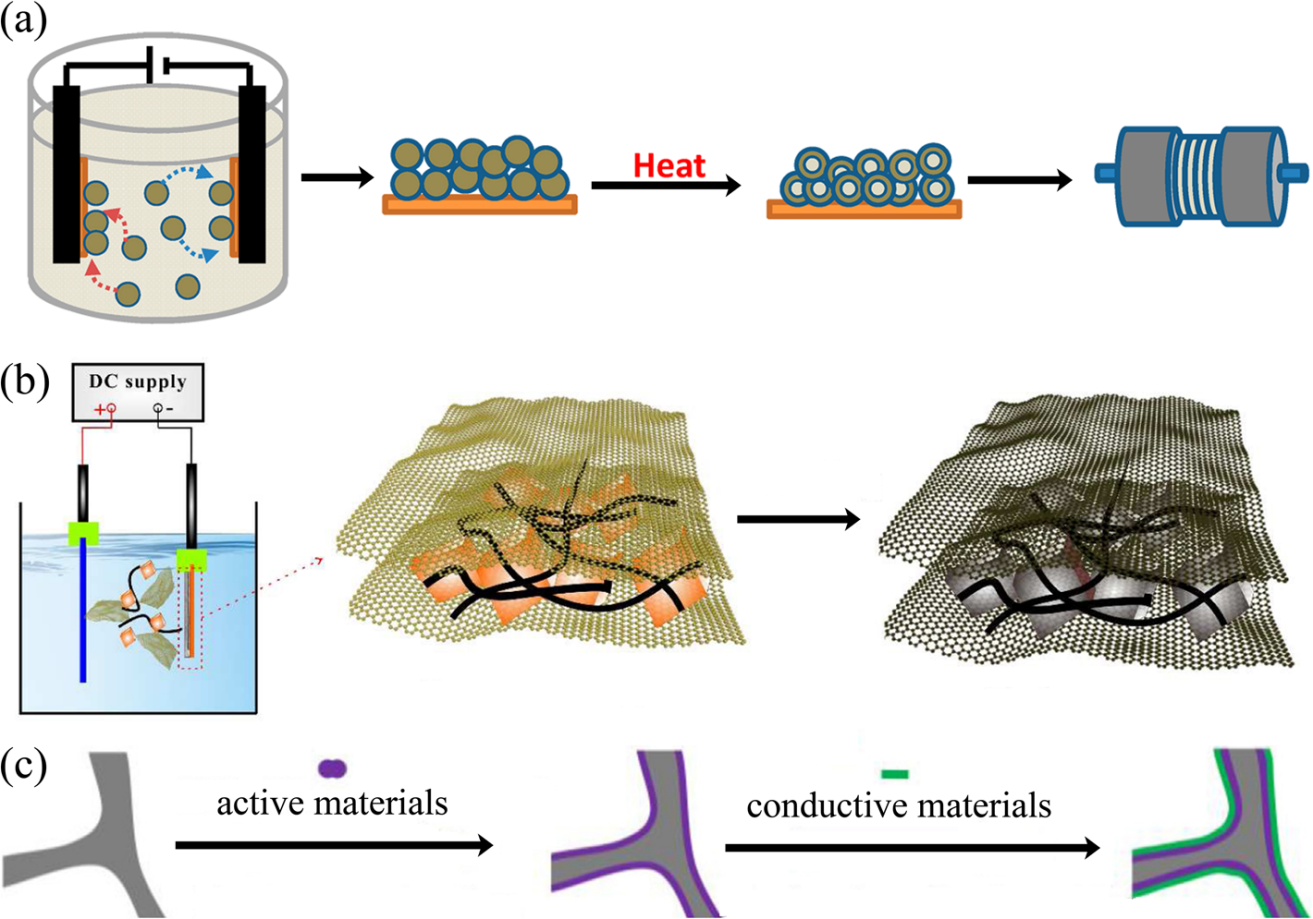
**a** Schematic of process for fabrication of binder-free, carbon-free film electrodes [101]. **b** Schematic fabrication process for the Fe_3_O_4_/CNTs/rGO composite electrode [102]. **c** Schematic illustration of the synthesis route for rGO/active materials/Ni foam [103]

Electrophoretic deposition (EPD) has been widely used as a surface coating and film preparation method. The deposition mechanism is that during the process, the charged particles with small sizes (need to disperse into the solution) in a suitable suspension migrate towards an electrode under an applied electric field (Fig. 6a, b). The morphology of the achieved film is significantly influenced by the electrolyte solution [104]. EPD has the advantages of low cost, simplicity, green, and controllable operation [105].

An electrode made by EPD shows better electrochemical performances than slurry-coated electrode. Robinson and coworkers proved that the Co_3_O_4_ nanoparticle films formed by EPD showed better adhesion and cycle performance than the electrode prepared by conventional methods (Fig. 6a). The EPD can provide a more effective mixed state between active materials and conductive additives [101]. It is worth noting that carbon nanotubes, graphene, and other carbon materials together with active materials can be deposited onto the current collector, which significantly improves the electron conductivity [106, 107]. Besides, the porous structure formed during the EPD process is crucial to accommodate the volume change during lithium-ion insertion and extraction. Zhao and coworkers demonstrated that the Si nanoparticle electrode prepared through EPD shows better electrochemical performance (Fig. 6b) [102, 108].

EPD is able to deposit surface layers composed of either active or inert materials. These layers serve as protective interfaces between the electrode and the electrolyte. For example, the reduced graphene oxide thin film deposited onto the surface of the electrode to improve the electrical conductivity and to buffer the volume changes during charge/discharge processes (Fig. 6c**)** [103].

## Physical Methods

### Electrospinning

**Fig. 7:**
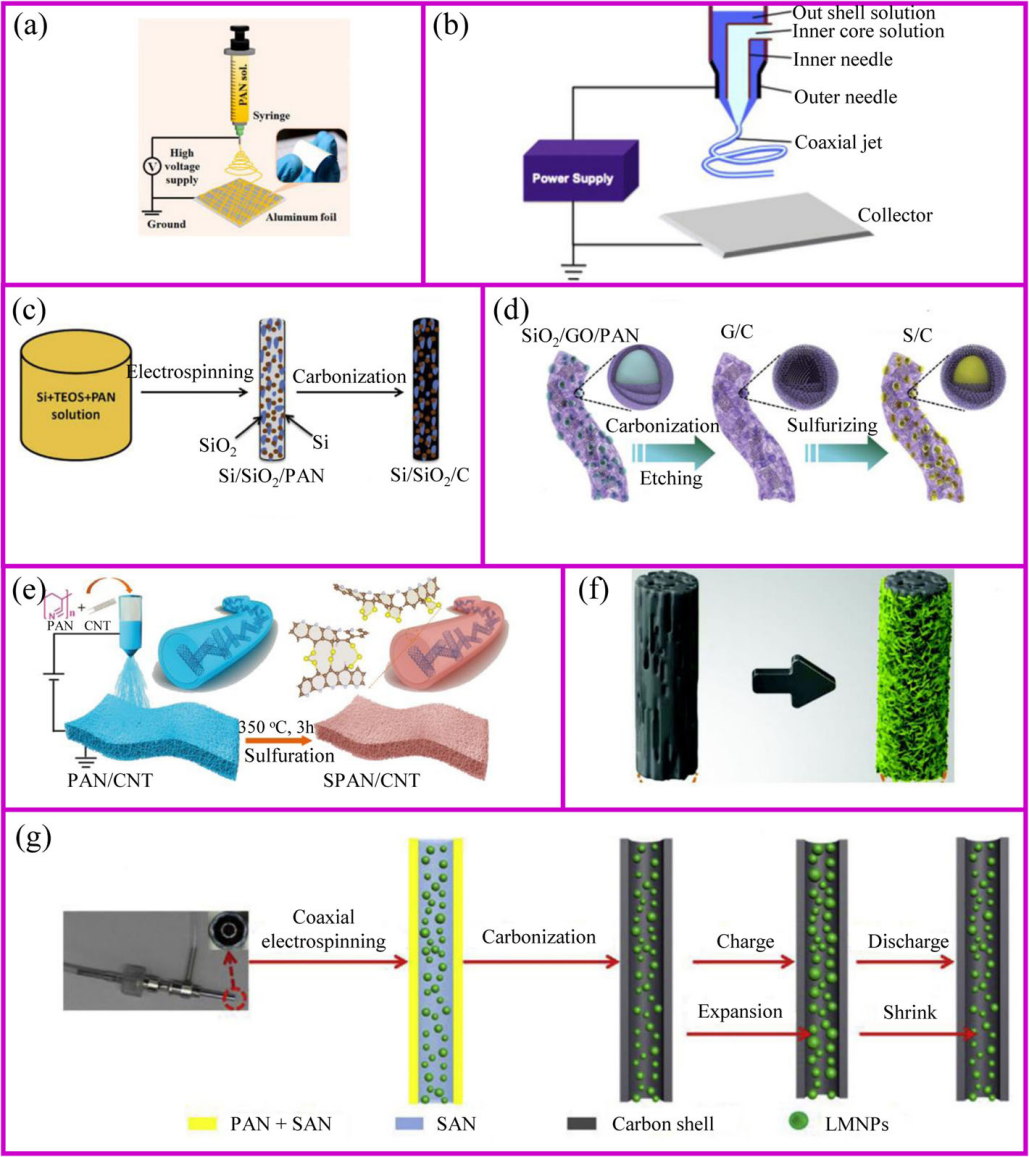
The schemes of **a** single axial and **b** coaxial electrospinning [111, 112]. **c** Inorganic fibers [113]. **d** Inorganic particles encapsulated carbon fibers [114]. **e** The modification of carbon fibers [115]. **f** Carbon fiber membrane with nanoparticles [38]. **g** Highly flexible carbon fiber membrane [116]

Electrospinning is a simple and popular technique to synthesize 1D nanostructures with fiber diameters ranged from tens of nanometers up to micrometers [109]. This preparation is difficult to achieve by the approaches mentioned above. This technique can produce polymers, organic, and inorganic composites with dense, hollow, or porous structures [110], from polymer solutions based on electrostatic forces [111]. An electrospinning unit generally consists of a syringe and a needle, a grounded collector, and a high-voltage supply, as shown in Fig. 7a, b [117]. During the electrospinning process, polymer solutions are loaded in the syringe and move into the needle to form a droplet. When a high voltage is applied between the needle and the collector, the electrostatic force at the surface of droplet would drive it to elongate to form a fiber. Finally, the solid polymer fibers would deposit onto the collector.

The polymer solutions and needle are the key points for the success of fiber fabrication. Polymer solution should reach the minimum viscosity for the formation of homogeneous fiber structure. The solvent of polymer should have a lower evaporation rate, which allows the polymer solidification after leaving the needle. The needle should be designed with coaxial structure to achieve hollow or core-shell fiber structure (Fig. 7b). For the coaxial electrospinning, the core and shell solutions should be adjusted to be immiscible or non-precipitable. Besides, during the electrospinning process, solution flow rates, voltage, temperature, distance from needle to the collector, and diameter of the needle have a huge influence on the fiber structure.

The obtained electrospun membrane needs further treatment to be a binder-free electrode. Carbon, ceramic, or metal nanofibers can be synthesized from the carbonization of electrospun fibers that contain polymer, metal salts, or metal atoms, respectively. Their composites such as metal/C and ceramic/C can be also obtained from their corresponding mixed precursors followed by a one-step or multi-step heat treatment. A wide range of electrospun materials have been investigated for LIBs including metal oxides (e.g., TiO_2_, Fe_2_O_3_, ZnO, NiO, CuO, LiCoO_3_, Li_4_Ti_5_O_12_, and LiMn_2_O_4_) [118, 119], hybrids [120] (e.g., SnO_x_/C, SiO_x_/C, Co_3_O_4_/C, SnO_x_/C, TiO_2_/C) [113, 121–130], and polymers (e.g., polyvinyl alcohol (PVA), PAN and PVP, poly(vinylidene fluoride-co-hexafluoropropylene) (PVDF-HFP), and polyethylene oxide (PEO)) [131].

Conventional electrospinning generally disperses metal salts and nanoparticles inside the fibers. However, the nanoparticles can adhere to the outside of the fibers as well (Fig. 7c). Lan, Yang, and coworker prepared 3D free-standing spider-web-like membranes with high mass loading of bismuth (Bi) nanoparticle clusters followed by carbonization in nitrogen gas [132]. The 3D Bi/C membrane provides good mechanical properties and stabilizes the Bi nanoparticles up to 200 cycles.

The architecture of fibers can be optimized to accommodate large volume changes and instability of the electrode materials during cycling process. The adjustment of the fiber structure can be started from either inside or outside of the fiber. The internal fiber can be regulated by the polymer solution and post-treatment, while the external fiber structure is controlled by post-treatment. When the polymer solution contains etchable materials, a porous fiber structure can be prepared after carbonization and template etching (Fig. 7d). This porous materials is capable of accommodating higher sulfur and suppressing the polysulfides shuttle effects [114]. The polymer can individually form an active material at the expense of flexibility self-standing property. This disadvantage can be addressed by additives. Liu et al. showed the PAN fibers with an appropriate amount of CNTs can still be self-standing after sulfurization [115]. The sulfur only exists in the form of Li_2_S_2_ and Li_2_S_3_ rather than polysulfides in the sulfurized PAN. Therefore, it shows ultra-stable cycling performance up to 1000 cycles (Fig. 7e).

Alternatively, the post-treatment of the surface of electrospun fibers is another way to prepare the high-performance binder-free electrode (Fig. 7f**)**. After carbonization, the three-dimensional conductive network is formed to provide good electronic conductivity. The fiber surface also provides a large number of sites for the growth of active materials with easy access to electrolyte [38]. Another post-treatment is to coat the nanofibers with a protective surface layer. Generally, the nanoparticles spinning out with the polymer solution is inevitably exposed at the surface of the fiber. This part of the material may fall off from fibers during the cycling process, so the surface coating is equivalent to the protection of the fiber [133].

In addition to polymer solution, the needle is also of importance to the fibers design. The core-shell composite nanofiber can be prepared by a dual nozzle coaxial electrospinning setup (Fig. 7g) [116]. This needle can achieve a great core-shell fiber structure. Besides, hollow fibers can be prepared by designing the inner and outer solutions. When the hollow fiber is filled with the active material, there is sufficient space to allow the volume to expand [112].

### Vacuum Filtration

**Fig. 8:**
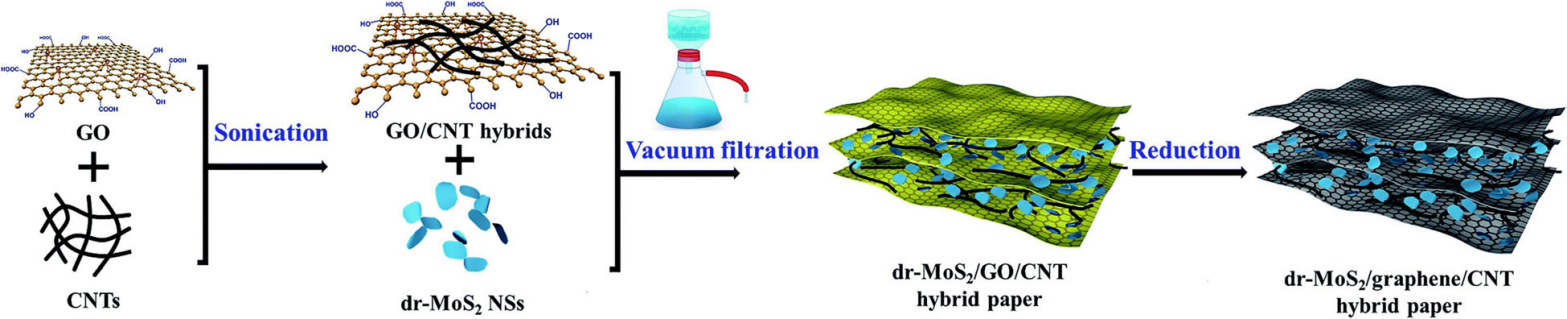
The scheme of vacuum filtration process [136]

The vacuum filtration method is a rapid manufacturing process to assemble different kinds of nanoscale materials into the macroscopic film for various applications. This process is low-cost, rapid, and efficient, which demonstrates a promising strategy for various functional films. 2D materials can be easily assembled into flexible self-standing paper-like materials, which can be directly used as flexible binder-free electrodes in energy storage devices [134, 135]. In general, the active materials are randomly dispersed between the supporting materials. Therefore, high mechanical strength and flexibility are preserved for the papers (Fig. 8) [136, 137].

The vacuum filtration features as the following strengths. Firstly, active materials can adhere on the conductive substrate, leading to the improvement of electron conductivity. For example, the electron conductivity of MoS_2_ can be largely improved; therefore, better rate performance can be obtained [138, 139]. Secondly, the large surface area is in favor of the contact between active materials and lithium ions, which facilitates the transportation of Li-ion. When the active material is added into the 2D material, the interlayer spacing becomes large; thus, the electrolyte can be immersed. The lithium ions are more accessible to the material; thereby, the interface impedance of material is reduced [140]. Thirdly, the effective material utilization is also facilitated by hindering the aggregation of 2D materials [141–143]. Lastly, the material agglomerations and electrode instabilities result from the huge volume change of active materials during Li insertion/extraction [144, 145]. Supporting sheets can absorb stress induced by volume expansion, similar to the role of elastic buffer [146, 147].

Different types of nanostructures can be assembled into 2D materials. For example, the nanoparticles, nanotubes, nanosheets, nanorods, etc. can fabricate into the graphene sheets [148]. When CNTs as additive are assembled into the nanosheets, the restacking of the nanosheets can be prevented, and the conductivity of ion and electron can be greatly increased [149]. The electrode chemical properties can be enhanced by coating or mixing active materials on other conductive materials and then assembling into 3D functional materials [150–152]. It is mainly attributed to the synergistic effects that 3D structure not only serves as a flexible scaffold for strains/stresses release and volume expansion, but also offers a three-dimensional conductive architecture with open channels for electron transfer and Li-ion diffusion. Besides, pre-protection of active materials is a way to improve material stability. The surface modified anode materials in graphene exhibit high capacities, long cycle-life, and excellent rate performance [153]. The Mn_2_P_2_O_7_-carbon in graphene electrode delivers a capacity of 585 mA h g^−1^ at a current density of 1000 mA g^−1^. When increasing the current density to 5000 mA g^−1^, a high capacity of 400 mA h g^−1^ can be remained even after 2000 cycles [153].

### Physical Vapor Deposition

**Fig. 9:**
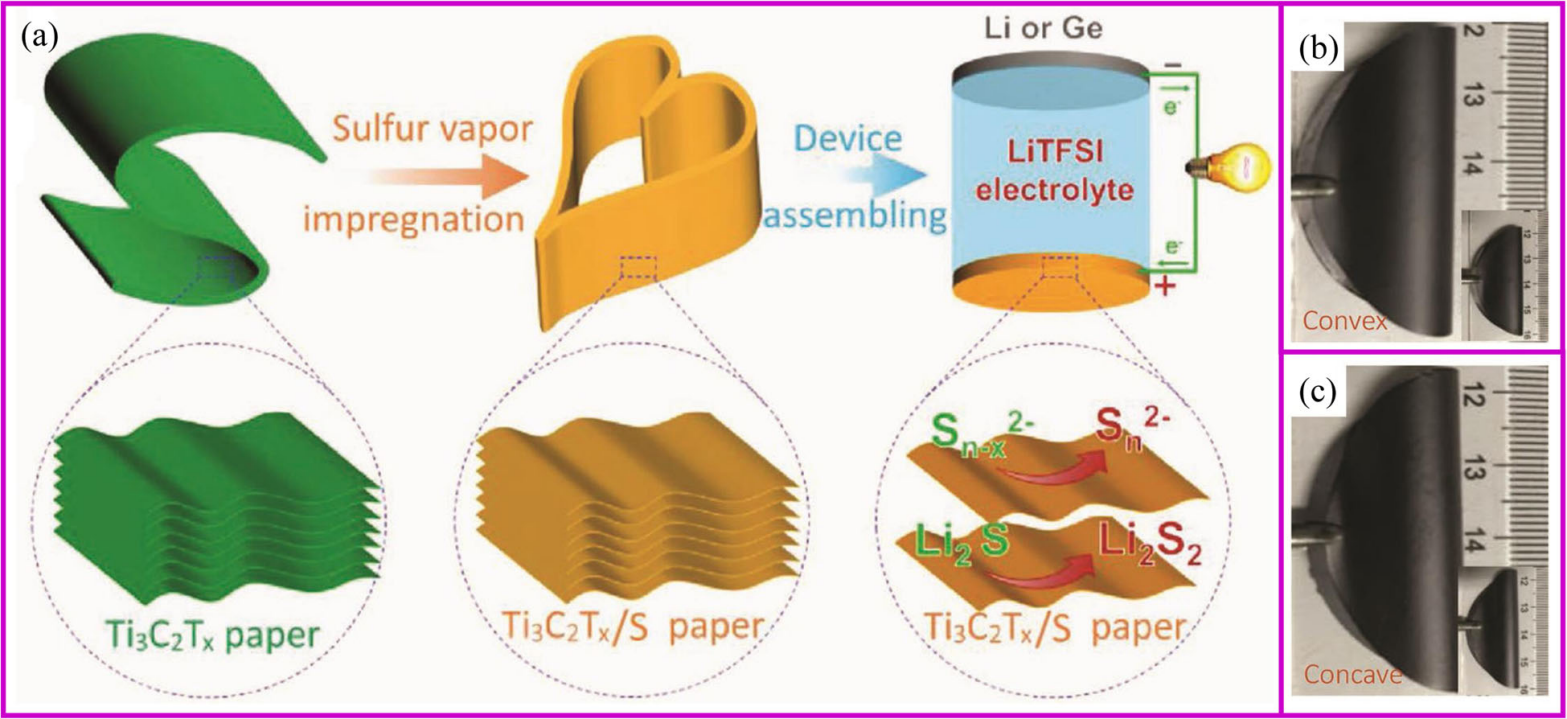
**a** The scheme of fabrication of robust, freestanding, and conductive Ti_3_C_2_T_x_/S paper. Photographs of freestanding Ti_3_C_2_T_x_/S paper when bending **b** convexly and **c** concavely, showing good mechanical flexibility similar to that of the pure Ti_3_C_2_T_x_ paper [158]

At certain temperature and airflow rate, the elemental vapor can be easily deposited onto the porous supporting materials [154–156]. Solid sulfur and red P nanoparticles are the typical materials, which can be deposited into porous carbon materials. The commercialization of sulfur as cathode materials is blocked by several intrinsic problems, including low electronic/ionic conductivity, large volumetric expansion, and shuttle effect of intermediate polysulfides (Li_2_S_x_ (4 ≤ *x* ≤ 8)). Particularly, the shuttle effect of polysulfides results in transport of sulfur from cathode to anode and the reaction with Li metal, which leads to significant capacity loss and safety issues. So far, the design of porous structure is the basic strategy to suppress the polysulfides shuttle effect, and sulfur vapor deposition is an effective way for the fabrication of S/C composite. It is an environmentally friendly, solvent-free method in which the sulfur powder undergoes a physical deposition process with no changes of chemical properties [157]. With proper absorbent in the structure, the shuttle effect of polysulfides can also be fixed. Recently, Yang, Zhang, and coworkers reported Ti_3_C_2_T_x_ paper is a good host for sulfur deposition (Fig. 9a). This Ti_3_C_2_T_x_ paper shows no cracks after 25 convexly and concavely bending cycles (Fig. 9b, c) [158]. Yu and coworkers [159] demonstrated porous carbon fibers encapsulated with red P shows high capacity of 2030 mAh g^−1^ at 0.1 C rate after 100 cycles. It is worth noting that physical vapor deposition (PVD) is only one of the procedures of immobilizing S or P onto carbon materials. Therefore, the most important research direction is how to design a porous conductive matrix.

## Application in Flexible Batteries

**Fig. 10:**
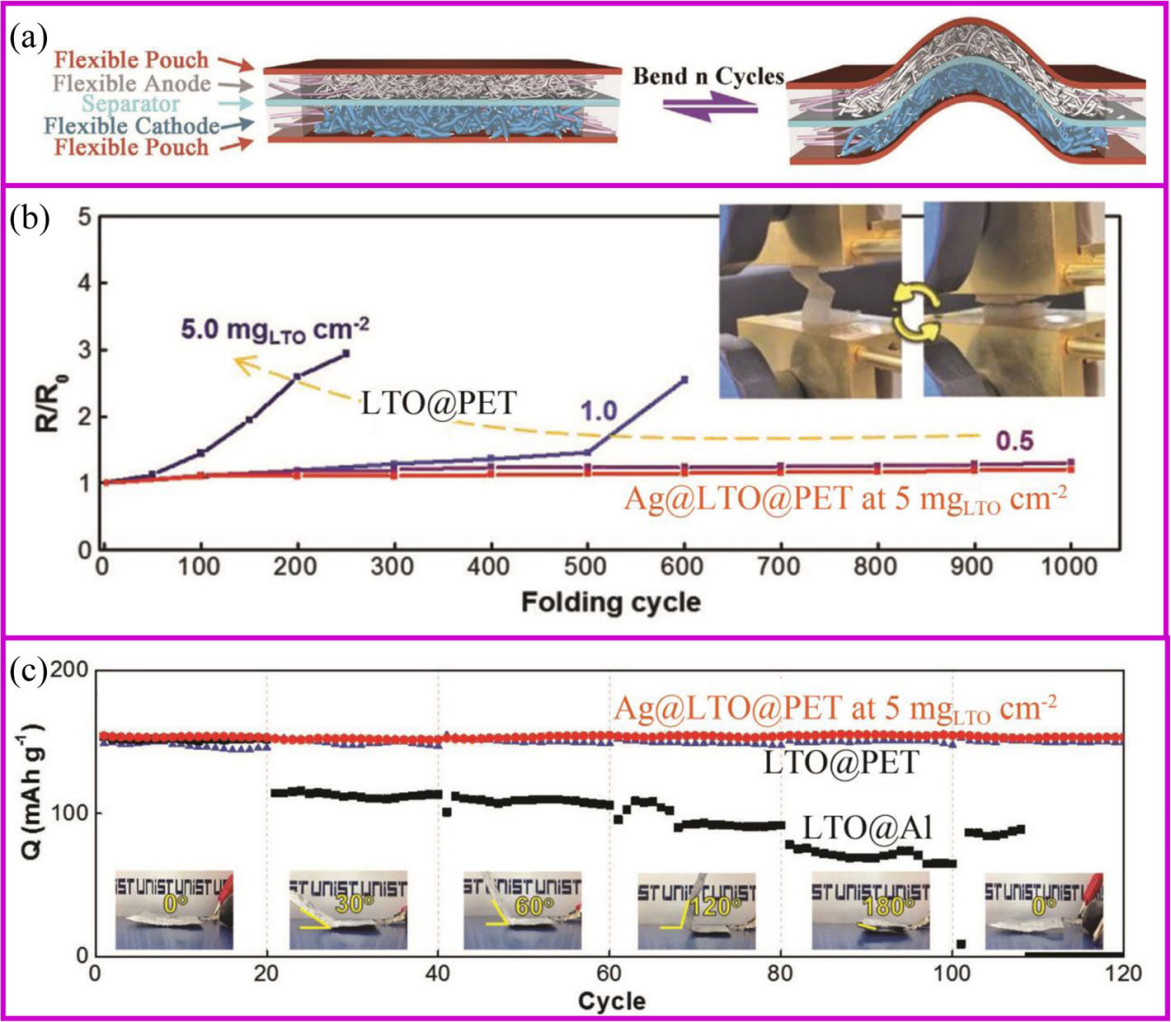
**a** Assembly and bending tests of flexible batteries with flexible electrodes [164]. **b** Electrical resistance change with folding cycles [165]. **c** Capacity retention of folded cells at different angles at 1 C [165]

Flexible devices, such as wearable displays, sensors, sportswear, mobile communication devices, rollup displays, and so on, are one of the important directions for intelligent and smart world [160]. The development of these new devices requires the power of a flexible battery system [161–163]. However, current advanced pouch and 18,650 cells cannot be used on flexible devices due to the rigid material properties. Each component of the flexible battery, such as electrodes, separator, and solid electrolyte, must be flexible (Fig. 10a) [164]. The conventional electrode is generally adhered to the metal foil by a coating method to physically bond the active material and the conductive agent. During repeated bending and folding, the active material separates from the current collector, ending up with deactivation. For example, the Li_4_Ti_5_O_12_ (LTO)-based electrode folded about 100 cycles would present the detachment of LTO from Al foil. The impedance of the electrode increases from the first fold, and the higher the active material loading, the faster the impedance increases (Fig. 10b). At the same time, the pouch cell bending 30° results in serious capacity fade (Fig. 10c).

**Fig. 11:**
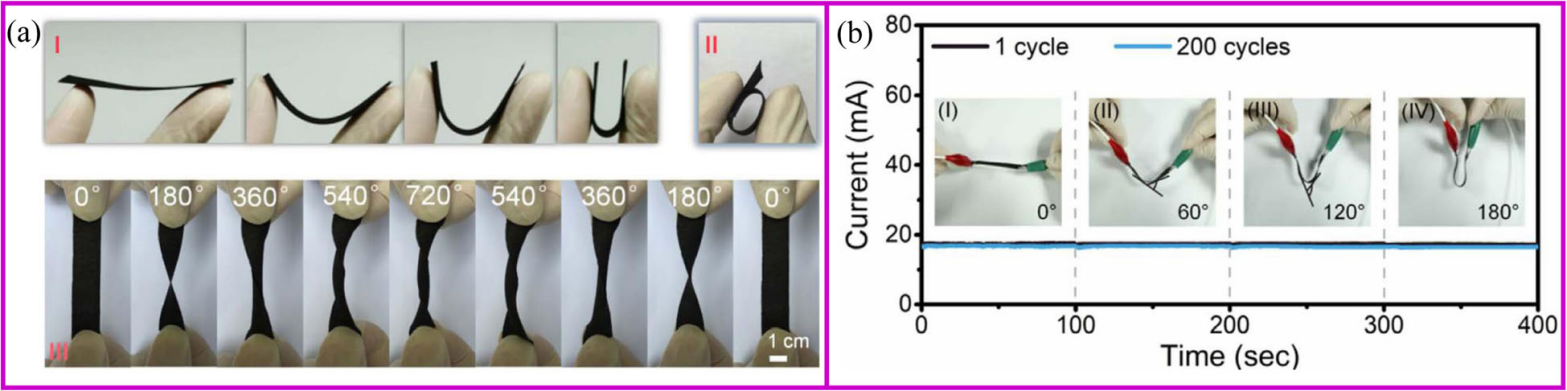
**a** Schematic illustration for the structural features of the flexible SnO_2_ nanosheets on flexible carbon cloth electrode during the folding (I), the rolling (II), and twisting (III) tests. **b** Current-time curves of the composite samples at various bending angles of the 1st and 200th cycles, and the inset images show the corresponding bending angles for measurement and photographs [166]

There are many strategies to fabricate flexible electrodes. Song et al. reported that coating LTO particles and Ag nano wires onto the polyethylene terephthalate (PET) web can greatly improve the electrode flexibility and stability. The electrical resistance of Ag@LTO@PET electrode does not change during 1000 folding cycles (Fig. 10b). Pouch-type Ag@LTO@PET-based half cells showed great cycling performance with little capacity decay when the electrode was bent at any angle (Fig. 11c) [165]. The most mature method is to fix the active material on a flexible substrate. As described in the “Introduction” section, the direct growth of the active material on the conductive substrate can improve battery energy density and rate performance. Herein, we take the carbon cloth and carbon materials as the example to show the application of binder-free electrodes in flexible devices.

Most carbon materials cannot be used in flexible electronics. For example, a binder-free electrode based on graphite paper can only maintain 25 cycles in a bent state [167]. Comparing with other carbon materials, carbon cloth with excellent flexibility and electrical conductivity is one of the most promising materials for the flexible battery application. Even after the surface modification of inorganic materials, carbon cloth still shows excellent flexibility. As shown in Fig. 11a, there are no apparent changes of the electrode after bending, rolling, twisting, folding, and crumpling tests. After the mechanical test, the active materials on the carbon cloth can maintain structural integrity. Also, after 200 bending cycles, the current value slightly decreases from 17.3 to 16.8 mA, which demonstrates great stability (Fig. 11b) [166].

**Fig. 12:**
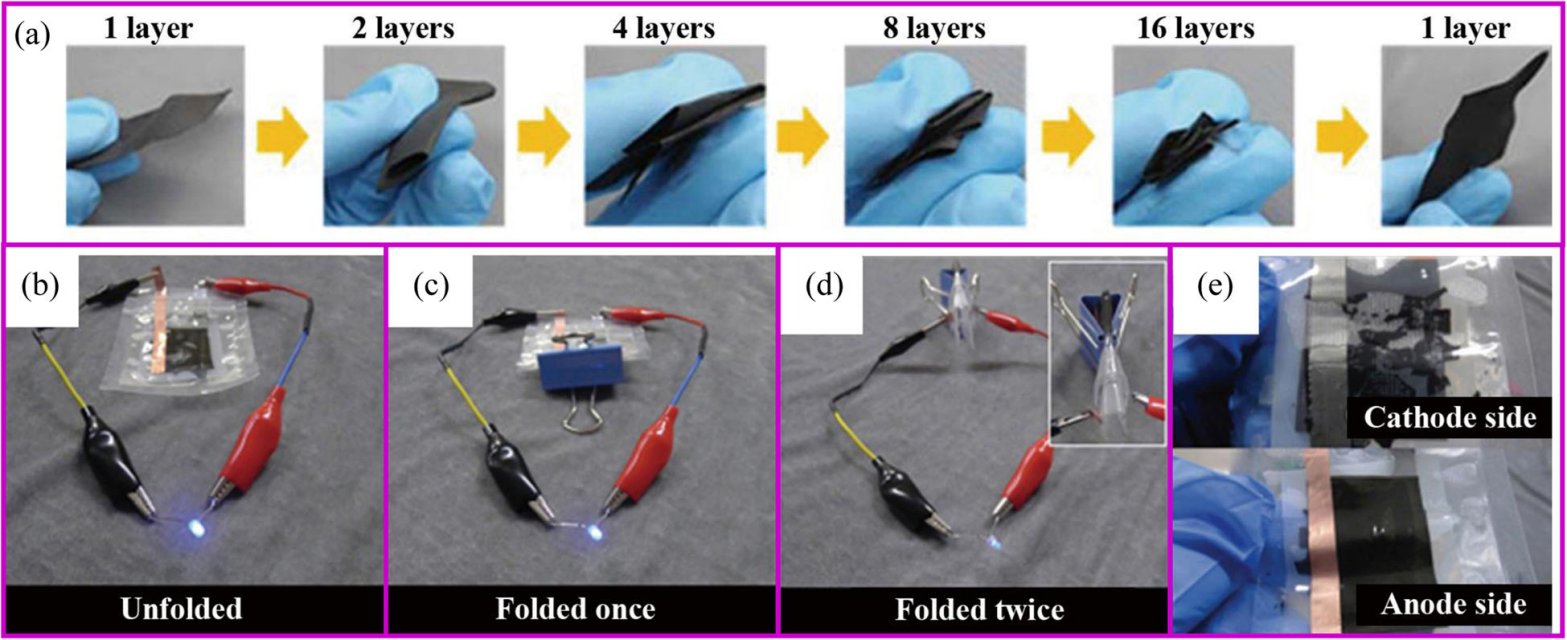
**a** Digital photographs of Zn(CH_3_COO)_2_-PAN film, which can be folded four times. LED lighting tests of a full battery when **b** flat, **c** folded once, and **d** folded twice; and **e** digital photographs of the electrode after the LED lighting test [130]

It is particularly difficult to synthesize flexible carbon materials. For example, the PAN film becomes much more brittle and fracture after carbonization, which is difficult to use in flexible batteries. The ideal carbon material, like the clothes we wear, bending and folding many times can still remain intact. The flexibility of the material can be greatly improved through reasonable design such as the addition of functional additives. Wang et al. reported that the carbonized PAN film with SiO_2_ filler can fully recover to its original state after repeated rolling or folding process [114]. When assembled into the pouch cell, it can withstand at different bending angles up to 180°. Yu et al. demonstrated that Zn(CH_3_COO)_2_ assists the uniform carbonization of PAN, which relieved the stress concentration [130]. The film obtained by this method can return to the initial state after folding four times (Fig. 12a). When assembled into the pouch cell, it can light the LED at any folding angle. When the pouch cell is disassembled, the binder-free electrode remains intact while the slurry-based electrode is completely destroyed (Fig. 12b–e).

## Conclusions

In conclusion, recent research progress on the preparation of binder-free electrodes for LIBs has been summarized. The fabrication methods focus on the chemical, physical, and electrical treatment, such as thermal treatment, hydrothermal treatment, CBD, ALD, CVD; vacuum filtration, electrospinning; and electrophoretic deposition, anodization, electrodeposition. Thermal treatment is the most commonly used chemical method to carbonize polymer for free-standing structure or decompose of the precursor of metallic oxide. The hydrothermal and CBD methods are very attractive due to accurate control of the size and morphology of nanomaterials. CBD and hydrothermal methods present in situ growth of active materials on the substrate through a chemical reaction. CVD is defined as the deposition of a gas carrier on a heated surface by a chemical reaction, while the ALD technique is a vapor phase chemical deposition process that is capable of producing high-quality nanoscale thin films in an atomic layer-by-layer manner. The vacuum filtration and electrospinning are the representative physical methods. The former is a physical manufacturing process to assemble different materials like nanoplatelets and nanoparticles into the macroscopic film. The latter can produce 1D nanoscale materials with fiber diameters ranged from tens of nanometers up to micrometers. The electrical method is a widely used technique to make coatings and thin films. However, it is not often used to prepare binder-free electrode. Among these methods, CVD and CBD are excellent ways to prepare silicon-based and sulfur-based materials, respectively.

The binder-free electrode shows better electrochemical performances than the traditional slurry system. The binder-free electrode can improve ionic and electronic transportation, cycling performance, and energy density of the electrodes. In addition, nanoscale materials are uniformly anchored on the supporting materials, which can effectively prevent the agglomeration of nanoparticles and mitigate the volumetric expansion during the repeated cycling process.

The conductive matrix plays a crucial role in the electrochemical properties and performances of the binder-free electrode. The ultra-flexible film has great potential to make a big breakthrough in the field of wearable and flexible devices. However, existing substrates are still unable to meet the requirements. The flexible device requires the binder-free electrode to bend and fold for numerous times with no damage and no separation from the substrate. According to current research process, ultra-flexible and ultra-stable carbon materials become the most promising candidate for next-generation flexible binder-free electrode.

Despite the difficulties, the future is expected. The uniform and large-scale growth of the active material on the conductive substrate is one of the necessary conditions for practical application. Fortunately, it is now possible to achieve. Practical applications need to consider the basic properties of the electrode in the battery, such as the initial Coulombic efficiency and voltage profiles. Therefore, the active materials for both anodes and cathodes should be carefully selected. For example, Si, Sn, or carbon materials serve as promising candidates for anode materials while the cathode materials may be selected from S matching with Li metal, or the existing Li metal oxides. In addition, flexible batteries can be achieved with all of flexible components, such as electrodes, separators, and electrolytes. Although these aspects have been studied for a long time, breakthrough is needed to facilitate the research progress.

## Data Availability

All data are fully available without restriction.

## Abbreviations

LIB: Lithium-ion batteries
PVDF: Polyvinylidene fluoride
ACF: Active carbon fiber
PAN: Polyacrylonitrile
rGO: Reduced graphene oxide
PVP: Polyvinylpyrrolidone
LBL: Layer-by-layer
GO: Graphene oxide
PDDA: Poly (diallyldimethylammonium chloride)
CNT: Carbon nanotube
CBD: Chemical bath deposition
CVD: Chemical vapor deposition
SEI: Solid electrolyte interface
Si NWs: Silicon nanowires
ALD: Atomic layer deposition
EPD: Electrophoretic deposition
PVA: Polyvinyl alcohol
PVDF-HFP: Poly(vinylidene fluoride-co-hexafluoropropylene)
PEO: Polyethylene oxide
Bi: Bismuth
PVD: Physical vapor deposition
LTO: Li_4_Ti_5_O_12_
PET: Polyethylene terephthalate
